# Clinical implication of COVID‐19 associated bradycardia

**DOI:** 10.1002/clc.23674

**Published:** 2021-06-19

**Authors:** Masaya Miyauchi, Teruhiko Imamura

**Affiliations:** ^1^ Second Department of Internal Medicine University of Toyama Toyama Japan

To the Editor

Kumar and colleagues performed a large‐scale multicenter retrospective study including more than 1000 COVID‐19 patients and found that bradycardia was prevalent and associated with higher mortality among them.^1^ Their findings are of great interest because infection and high fever are, in general, associated with tachycardia. Several concerns have been raised.

To further clarify the mechanism of COVID‐19 associated bradycardia, it is of great interest to investigate the type of bradycardia: sinus bradycardia, AV block, or atrial fibrillation. Noncardiac causes should be carefully excluded, including sedation, conduction‐suppressive medications, and hypothyroidism.

The causality between bradycardia and mortality remains unclear. Bradycardia might be a marker of the advanced stage of COVID‐19 or a direct cause of death. Bradycardia might be more prevalent in patients with COVID‐19 associated myocarditis with incremental troponin level. On the other hand, bradycardia might cause low cardiac output accompanying cardiogenic shock. If bradycardia is harmful, aggressive intervention to bradycardia, including pacemaker therapy, would be beneficial. In their study, was the dominant cause of death in patients with bradycardia respiratory failure or heart failure?

## DISCLOSURE OF INTEREST

Teruhiko Imamura receives grant support from JSPS KAKENHI: JP20K17143.

## Data Availability

This manuscript does not include any data.
